# Immune Gene Expression Profiling in Individuals with Turner Syndrome, Graves’ Disease, and a Healthy Female by Single-Cell RNA Sequencing: A Comparative Study

**DOI:** 10.3390/cells14020093

**Published:** 2025-01-10

**Authors:** Soo Yeun Sim, In-Cheol Baek, Won Kyoung Cho, Min Ho Jung, Tai-Gyu Kim, Byung-Kyu Suh

**Affiliations:** 1 Department of Pediatrics, Seoul St. Mary’s Hospital, College of Medicine, The Catholic University of Korea, Seoul 06591, Republic of Korea; sooyeunsim8@gmail.com (S.Y.S.); suhbk@catholic.ac.kr (B.-K.S.); 2 Catholic Hematopoietic Stem Cell Bank, College of Medicine, The Catholic University of Korea, Seoul 06591, Republic of Korea; 3 Department of Pediatrics, St. Vincent’s Hospital, College of Medicine, The Catholic University of Korea, Seoul 16247, Republic of Korea; 4 Department of Pediatrics, Yeouido St. Mary’s Hospital, College of Medicine, The Catholic University of Korea, Seoul 07345, Republic of Korea; jmhpe@catholic.ac.kr; 5 Department of Microbiology, College of Medicine, The Catholic University of Korea, Seoul 06591, Republic of Korea; kimtg@catholic.ac.kr

**Keywords:** Turner syndrome, X chromosome inactivation, autoimmunity, gene expression, single-cell RNA analysis

## Abstract

Turner syndrome (TS) can be determined by karyotype analysis, marked by the loss of one X chromosome in females. However, the genes involved in autoimmunity in TS patients remain unclear. In this study, we aimed to analyze differences in immune gene expression between a patient with TS, a healthy female, and a female patient with Graves’ disease using single-cell RNA sequencing (scRNA-seq) analysis of antigen-specific CD4(+) T cells. We identified 43 differentially expressed genes in the TS patient compared with the healthy female and the female patient with Graves’ disease. Many of these genes have previously been suggested to play a role in immune system regulation. This study provides valuable insights into the differences in immune-related gene expression between TS patients, healthy individuals, and those with autoimmune diseases.

## 1. Introduction

Turner syndrome (TS) is one of the two relatively common forms of female X chromosome aneuploidy, marked by distinctive physical traits such as short stature, gonadal dysgenesis, neck-webbing, and cardiovascular and renal malformation, while in many cases an increased incidence of autoimmune disease is also observed [1,2]. A recent Danish nationwide study reported a rising incidence of TS, suggesting a birth incidence of 1 in 1700 live-born females [3]. In South Korea, the TS prevalence was 7.84 per 100,000 females and a shorter life expectancy of women with TS than the general female population was reported [4]. The etiology behind the phenotypic variability in Turner syndrome is complex and not fully understood. The mechanism by which the absence of an X chromosome determines the characteristics of TS is poorly understood [5]. Traditionally, the TS phenotype has been explained by X chromosome monosomy, where the missing or structurally altered second X chromosome results in altered regulation of gene expressions that are normally present in two copies in individuals with two intact X chromosomes [6]. However, recent studies have suggested that X chromosome variation not only affects the expression level of genes of the X chromosome, but also impacts the expression of genes on other chromosomes [7]. Hence, while the deleted gene on the missing second X chromosome may individually influence the TS phenotype, the additive effects of genes on other chromosomes might also play a role [8]. These effects could result from changes in gene expression regulation, potentially prompted by epigenetic factors, contributing to the phenotypic variability of the TS phenotype [5].

Graves’ disease (GD) predominantly affects females, drawing significant attention to the underlying sexual dimorphism in immune response [9,10]. The adaptive immune response plays an important role in the pathogenesis of GD, characterized by a Th2-biased response and a strong focus on antibody production [11,12]. T cells are vital components of the adaptive immune response within the lymphoid system, constituting approximately three-quarters of all lymphocytes. CD4(+) T cells, specifically, play a critical role in pathogen clearance, autoimmune disease regulation, and the elimination of pathogenic cells [13]. Furthermore, distinct CD4(+) T cell subsets produce different cytokine profiles, protecting against various pathogens and mediating immune pathologies. *GATA3*, a key transcription factor, is involved in the Th2 differentiation, leading to eosinophils, mast cells, and IgE-producing B cell activations [14]. CD4(+) T cells have been associated with the pathogenesis of various autoimmune diseases, including thyroid disorders such as autoimmune thyroid disease (AITD) [15]. Some suggest that the impact of X chromosome inactivation escape and skewing toward autoimmune diseases could be explained by the reactivation and loss of mosaicism hypothesis [16]. In this genome-wide association study (GWAS), *GPR174/ITM2A* on the X chromosome was recognized as the second most potent AITD-susceptible gene following *HLA* [17].

Autoimmune diseases represent a significant health concern in Turner syndrome. The most frequently reported autoimmune risk in TS patients is thyroid dysfunction, with thyroperoxidase (Anti-TPO) antibodies detected in 48% of TS patients, compared with 13% in the general population [18,19,20]. In TS, an increased relative risk of diabetes has been reported, demonstrating its significant health burden [21]. The X chromosome harbors approximately 1000 genes, including those encoding receptors and associated proteins, immune-related proteins, as well as factors involved in transcriptional and translational regulation. Defects in certain X chromosome-linked genes are associated with various immune system abnormalities, including issues with immunoglobulin production and the regulation of T cell and B cell functions. These abnormalities have been linked to the clinical characteristics of Turner syndrome, highlighting their potential to induce specific features of TS [22,23]. Bianchi et al. proposed that haploinsufficiency of immune-related genes on the X chromosome may contribute to the development of autoimmune diseases [24]. However, previous studies on altered gene expressions in TS patients have reported controversial results [25]. While some suggest that genetic variations in numerous immune-related genes on the X chromosomes are linked to sex-based differences in immune response and higher prevalence of certain diseases in females, others have argued for the reactivation and loss of mosaicism hypothesis [16,26,27].

Despite breakthroughs and advances in technology, our knowledge of sex chromosome aneuploidies and the genetic regulations of Turner syndrome remains limited. To uncover key immune gene expression patterns associated with each condition, we performed single-cell RNA sequencing (scRNA-seq) analysis to compare CD4(+) T cell immune profiling among three individuals: one with complete monosomy X Turner syndrome (45, X), representing haploinsufficiency of the X chromosome; a healthy female (46, XX); and a female with Graves’ disease (46, XX), serving as a representative of a normal karyotype female with an autoimmune condition.

## 2. Materials and Methods

### 2.1. Subjects and Human Blood Samples

This study was approved by the Institutional Review Board (IRB) of the Catholic University of Korea (IRB Number: KC23TISI0193). All participants provided written informed consent prior to participation in this study. Peripheral blood mononuclear cell (PBMC) samples were obtained from one healthy female (HF, 46, XX) volunteer, one patient diagnosed with Turner syndrome (TS, 45, X), and one patient diagnosed with Graves’ disease (GD, 46, XX). TS was diagnosed based on chromosome analysis. GD was diagnosed through thyroid assessment, including high radioactive iodine intake, clinical symptoms of hyperthyroidism, positive thyroid stimulating hormone (TSH) receptor antibodies, and elevated thyroid hormone levels. PBMCs were isolated using Ficoll-Hypaque (GE Healthcare, Chicago, IL, USA). An AutoMacs Pro separator (Miltenyi Biotec, Bergisch Gladbach, Germany) was used to isolate CD4(+) T cells via magnetic microbeads. Flow cytometric analysis confirmed the purity of the CD4(+) T cells [28].

### 2.2. Single-Cell Library Preparation

CD4(+) T cells gated single-cell RNA sequencing was performed for peripheral blood mononuclear cells isolated from blood samples of a patient with Turner syndrome (45, XO), a heathy female (46, XX), and a female with Graves’ disease (46, XX). The Chromium Single Cell Gene Expression Solution with Chromium Single Cell 3′ GEM, Library and Gel Bead Kit v2 (10xGenomics, Pleasanton, CA, USA) was used for library preparation and processing following the manufacturer’s specification. The 10x Genomics system with rapid droplet-based encapsulation of single cells uses a gel bead in emulsion (GEM) approach. Each GEM contains a single cell, a gel bead with barcoded oligonucleotides, and reverse transcription regents. Isolated CD4 T (+) cells were suspended in a master mix solution. A LUNA-FL^tm^ Automated Fluorescence Cell Counter (Logos Biosystems, Anyang, Republic of Korea) was used to select cells with viability between 70% and 90%. These cells were then loaded into a well of the channel of a Single Cell A chip. Next, gel beads and partitioning oil were added to another channel well. A Chromium Controller (10x Genomics) lysed single cells and dissolved gel beads to release identically barcoded oligonucleotides for cDNA synthesis. By dividing a thousand of the cells into a nanoliter-scale GEM, all DNA molecules shared a 10-fold barcode. Each cell and transcript were uniquely barcoded with a unique molecular identifier (UMI). The cDNA used for sequencing was generated according to the manufacturer’s protocol. Transcripts were then amplified to the barcoded cDNA using a thermocycler (Macrogen Inc., Seoul, Republic of Korea). The products were purified and concentrated by PCR to generate a final cDNA library. The purified libraries were quantified using qPCR and assessed using TapeStation 4200 (Agilent Technologies, Inc., Santa Clara, CA, USA). Libraries were sequenced on an Illumina HiSeq 4000 (Macrogen Inc.) according to the read length [29].

### 2.3. Data Collection

Cell Ranger Single Cell v2.1.1 software (10x Genomics), an analysis pipeline, was used for processing the sequenced data, including barcode processing, UMI, gene counting, and mapping [30]. Using Cell Ranger’s mkfastq, raw BCL files were demultiplexed into FASTQ files [31]. FASTQ files were transferred to BAM (Binary Alignment Map) format. Differential gene expression between cell groups was analyzed using the negative binomial exact test (sSeq method) implemented in the Cell Ranger program. Cell Ranger compares the identified cell clusters to each other to determine genes highly expressed in a cluster with respect to other clusters. A total of 13,739 cells were processed in Og-NSCs (3D_cellRanger), with an average 39,181 reads and 2353 genes per cell. Only reads uniquely mapped to the transcriptome were included for UMI counting in the Cell Ranger, with the UMI count per cell serving as the unit of gene expression. The Loupe Browser (10x Genomics) was further used for clustering and visualization of the dataset. The Louvain algorithm was used to identify the cell type and clustering [32]. The cells were further clustered by Th2 for subclassification analysis [14,33]. The clustering algorithm partitions the pre-computed neighbor graph into modules, which are clusters of cells. Cells with similar gene expression profiles were placed close to each other while those with differences were placed further apart. The estimated number of cells was performed by ‘cellranger count’. The number of barcodes associated with cell-containing partitions was estimated based on the distribution of barcode UMI count. Briefly, the cellranger count fetches FASTQ files from the cellranger mkfastq and performs sorting, filtering, barcode counting, and UMI counting. Using a chromium cellular barcode, a feature barcode matrix is generated, clusters are determined, and gene expression analysis is performed. The count pipeline can receive input through multiple sequencing runs on the same GEM well. The selector count also processes feature barcode data along with gene expression readings [34].

### 2.4. Statistical Analysis

Gene expression levels were normalized and adjusted using the global-scaling normalization method ‘LogNormalize’ in Seurat v3.1 [35,36]. This method normalizes expression measurements for each cell by dividing by the total expression, multiplying by a scale factor (default: 10,000), and applying a log transformation to the resulting values. Uniform Manifold Approximation and Projection (UMAP) analysis was employed for dimension reduction and batch correction was performed using Anchors and Canonical Correlation Analysis (CCA) in Seurat 3.1. Differential expression analysis was conducted using the Wilcoxon rank-sum test, with *p*-values adjusted for multiple comparisons using the Benjamini–Hochberg correction in the Loupe Browser [37,38].

## 3. Results

### 3.1. Participants

**Figure 1:**
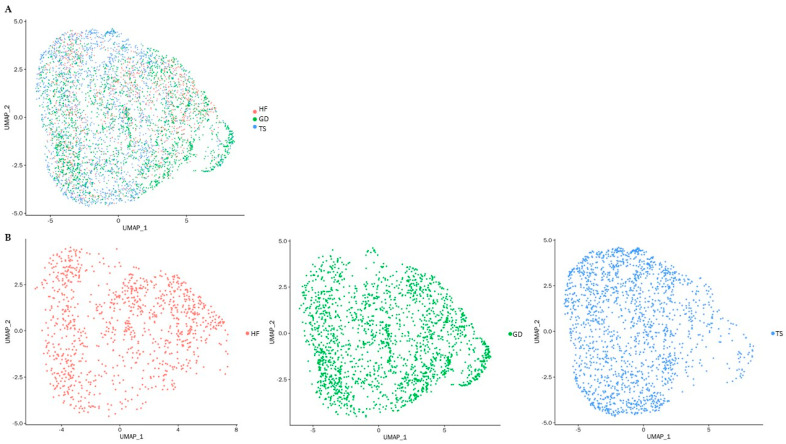
UMAP presentation of single-cell RNA sequencing of PBMCs. Each dot represents one of the CD4(+) T cells; (**A**) UMAP plot colored by study subjects: cells are represented based on three different groups indicated in red, green, and blue, respectively; (**B**) UMAP plot of individual study subjects: CD4(+) T cells of a healthy female (HF) are represented in red; CD4(+) T cells of a patient with Graves’ disease (GD) are represented in blue; CD4(+) T cells of a patient with Turner syndrome (TS) are represented in green.

One healthy female (HF, 46, XX) volunteer, one patient diagnosed with Turner syndrome (TS, 45, X), and one patient diagnosed with Graves’ disease (GD, 46, XX) donated peripheral blood mononuclear cell (PBMC) samples for this study. The baseline characteristics of study subjects are presented in Table 1. PBMCs of study subjects were further analyzed based on CD4(+) T cell distributions (Figure 1).

### 3.2. Single-Cell Gene Expression Differences Between Turner Syndrome and Healthy Female

CD4(+) T cells gated single-cell RNA-sequencing was used to conduct a detailed comparison of gene expression patterns between a Turner syndrome patient, a healthy female, and a female with Graves’ disease. A total of 718 differentially expressed genes (DEGs, *p* < 0.05) between TS and a healthy female control were identified. Table 2 represents the top 30 most significantly differentially expressed genes, sorted by the level of significance. Subsequent analysis of CD4(+) T cells was carried out by gating the results with *GATA3* expression to distinguish significant gene expressions in Th2 cells in a patient with TS compared with an HF. The top 30 significant genes are shown in Table 3.

### 3.3. Single-Gene Expression Differences Between Turner Syndrome and a Female with Graves’ Disease

There were 1574 differentially expressed genes (DEGs, *p* < 0.05) between the Turner syndrome patient and the female patient with Graves’ disease. The top 30 most significantly differentially expressed genes are shown in Table 4. Significant differences in single-cell gene expression profiles of Th2 cells in a patient with TS compared with a patient with GD were identified by gaiting the results with GATA3 expression (Table 5).

### 3.4. Differentially Expressed Genes in Turner Syndrome Compared with Healthy Female and Graves’ Disease Female

**Figure 2:**
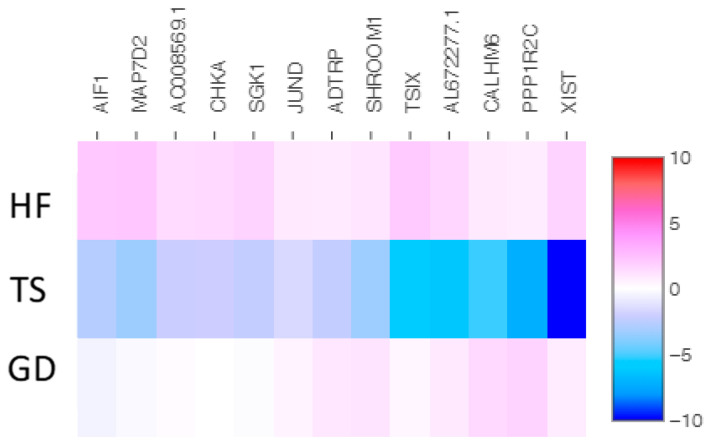
The heatmap of thirteen shared down-expressed genes in a patient with TS compared with both an HF and a patient with GD. Values are presented on a Log2 scale.

**Figure 3:**
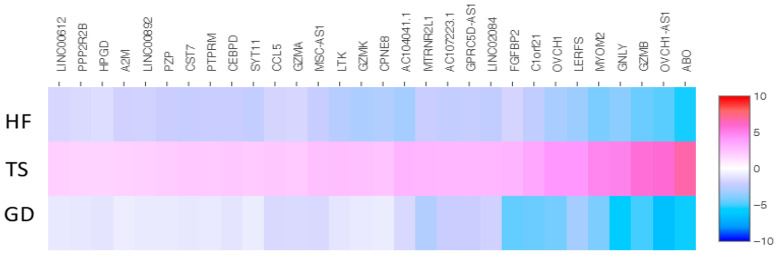
The heatmap of thirty shared up-expressed genes in a patient with TS compared with both an HF and a patient with GD. Values are presented on a Log2 scale.

We identified 43 overlapping genes (30 up-expressed and 13 down-expressed genes) in Turner syndrome compared with a healthy control female and a female with Graves’ disease (Figure 2 and Figure 3). *XIST*, *PPP1R2C*, *CALHM6*, *AL672277.1*, *TSIX*, *SHROOM1*, *ADTRP*, *JUND*, *SGK1*, *CHKA*, *AO008569.1*, *MAP7D2*, and *AIF1* were down-expressed genes in TS compared with both the healthy female and the female patient with Graves’ disease. The following 30 overlapping genes were up-expressed in TS: *ABO*, *OVCH1-AS1*, *GZMB*, *GNLY*, *MYOM2*, *LERFS*, *OVCH1*, *C1orf21*, *FGFBP2*, *LINC02084*, *GPRC5D-AS1*, *AC107223.1*, *MTRNR2L1*, *AC104041.1*, *CPNE8*, *GZMK*, *LTK*, *MSC-AS1*, *GZMA*, *CCL5*, *SYT11*, *CEBPD*, *PTPRM*, *CST7*, *PZP*, *LINC00892*, *A2M*, *HPGD*, *PPP2R2B*, and *LINC00612*.

**Figure 4:**
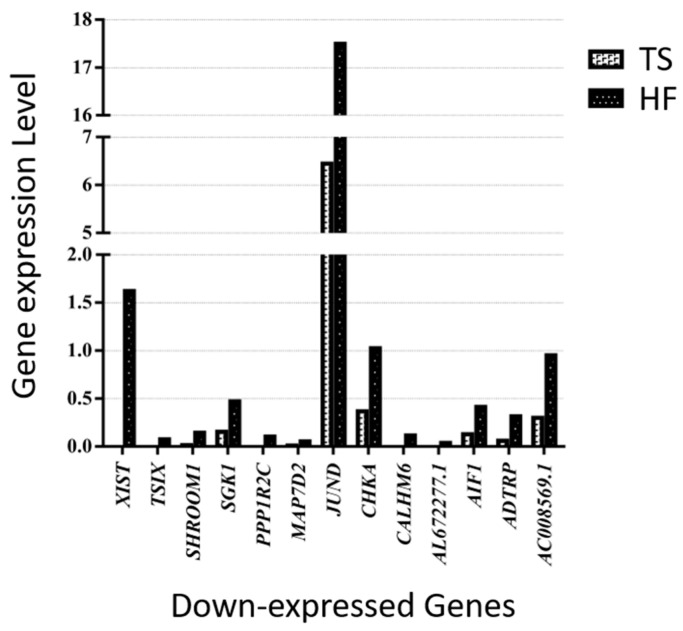
Thirteen down-expressed genes in a patient with TS compared with an HF.

**Figure 5:**
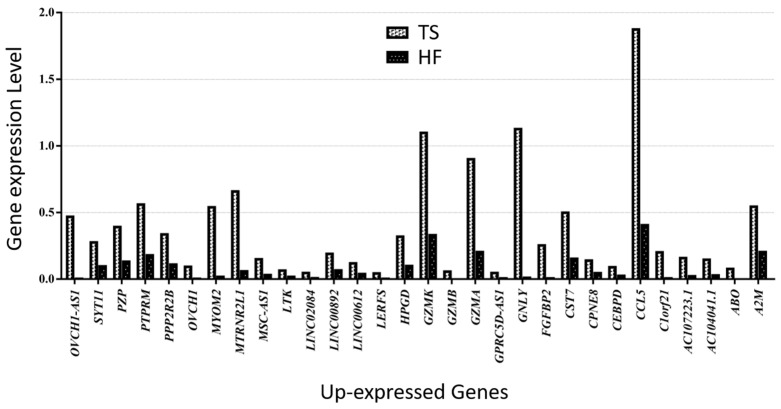
Thirty up-expressed genes in a patient with TS compared with an HF.

Of those overlapping genes, the following 13 genes were down-expressed in the Turner syndrome patient compared with the healthy female control: *XIST*, *TSIX*, *SHROOM1*, *SGK1*, *PPP1R2C*, *MAP7D2*, *JUND*, *CHKA*, *CALHM6*, *AL672277.1*, *AIF1*, *ADTRP*, and *AC008569.1* (Figure 4). On the contrary, *OVCH1-AS*, *SYT11*, *PZP*, *PTPRM*, *PPP2R2B*, *OVCH1*, *MYOM2*, *MTRNR2L1*, *MSC-AS1*, *LTK*, *LINC02084*, *LINC00892*, *LINC00612*, *LERFS*, *HPGD*, *GZMK*, *GZMB*, *GZMA*, *GPRC5D-AS1*, *GNLY*, *FGFBP2*, *CST7*, *CPNE8*, *CEBPD*, *CCL5*, *C1orf21*, *AC107223.1*, *AC104041.1*, *ABO*, and *A2M* were up-expressed genes in the TS patient (Figure 5).

**Figure 6:**
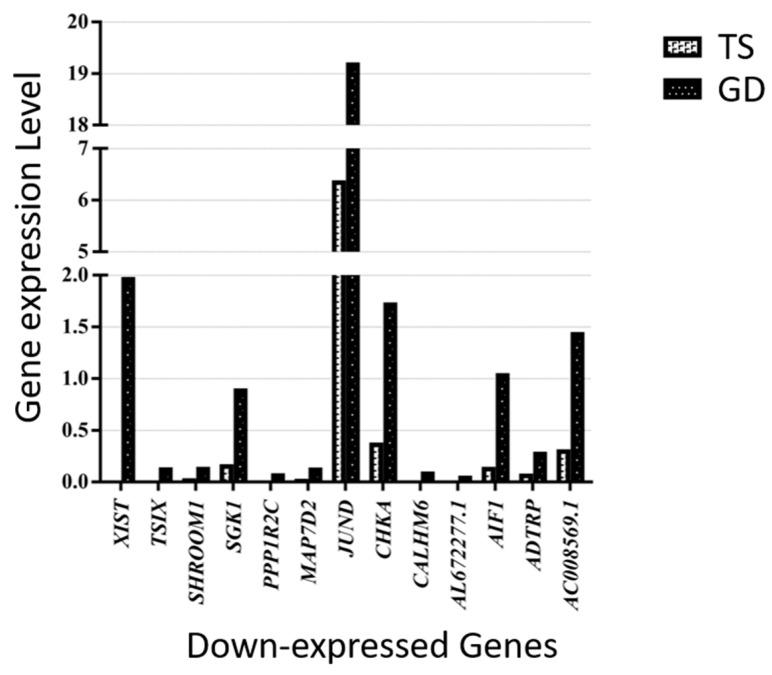
Thirteen down-expressed genes in a patient with TS compared with a female patient with GD.

**Figure 7:**
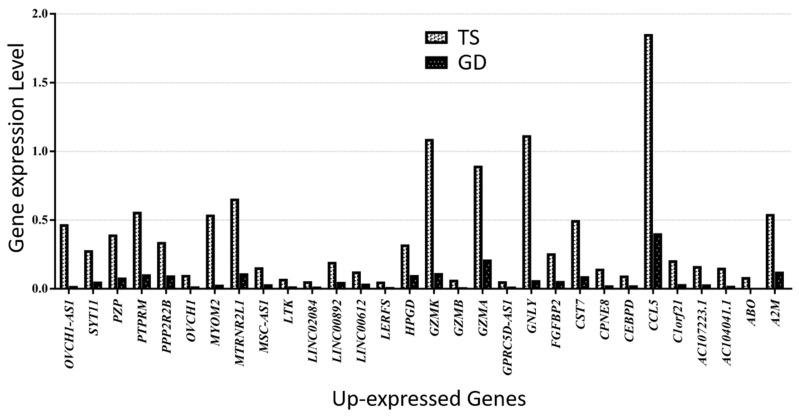
Thirty up-expressed genes in a patient with TS compared with a female patient with GD.

Similarly, 13 genes (*XIST*, *TISX*, *SHROOM1*, *SGK1*, *PPP1R2C*, *MAP7D2*, *JUND*, *CHKA*, *CALHM6*, *AL672277.1*, *AIF1*, *ADTRP*, and *AC008569.1*) were down-expressed in the Turner syndrome patient compared with the Graves’ disease patient (Figure 6). *OVCH1-AS1*, *SYT11*, *PZP*, *PTPRM*, *PPP2R2B*, *OVCH1*, *MYOM2*, *MTRNR2L1*, *MSC-AS1*, *LTK*, *LINC02084*, *LINC00892*, *LINC00612*, *LERFS*, *HPGD*, *GZMK*, *GZMB*, *GZMA*, *GPRC5D-AS1*, *GNLY*, *FGFBP2*, *CST7*, *CPNE8*, *CEBPD*, *CCL5*, *C1orf21*, *AC107223.1*, *AC104041.1*, *ABO*, and *A2M* were the genes up-expressed in the TS patient compared with the Graves’ disease patient (Figure 7).

## 4. Discussion

To the best of our knowledge, this is the first study to apply CD4(+) T cells gated single-cell RNA sequencing of peripheral blood mononuclear cell samples to identify differentially expressed genes in a Turner syndrome patient compared with a healthy female and a female patient with Graves’ disease. Graves’ disease, a female-predominant condition, exhibits adaptive immune response biased towards CD4(+) T cells. Therefore, in this study CD4(+) T cell immune profiling for an individual with compete monosomy X Turner syndrome (45, X), a female patient with GD (46, XX with autoimmune disease) and a healthy female (46, XX) was conducted. The thyroid function of the TS patient and the healthy female was within the normal range, whereas the GD patient showed initial diagnostic results consistent with hyperthyroidism. In the overall clustering analysis with UMAP plots, we presented the CD4(+) T cells from all three subjects and identified significant differences in gene expression profiles across the groups. The top 100 significantly differentially expressed genes are provided in the Supplementary Materials (Supplementary Tables S1–S4). Among these, 43 genes were differentially expressed in TS compared with both the healthy female and the patient with Graves’ disease, with 30 genes being up-expressed and 13 genes being down-expressed. Notably, *XIST*, a gene known to initiate X chromosome inactivation to balance the X chromosome-related gene expression, was significantly down-expressed in TS compared with both the GD patient and the healthy female, consistent with previous findings [24,39,40,41]. These results further support that the observed differences are specific to TS, underscoring the unique transcriptional profile of monosomy X. Additionally, they provide insights into the potential implications of these gene expression changes for immune system function when compared with healthy individuals and those with autoimmune disorders.

A recent guideline updated in 2024 reported that women with Turner syndrome face a significantly increased risk of autoimmunity, with a 61% lifetime prevalence [21]. Various studies have documented immunological alterations in TS patients, suggesting that the rise in autoimmunity shown in TS is multifactorial. In 2020, Wang et al. conducted a bioinformatic analysis of the Gene Expression Omnibus (GEO) dataset GSE46687, which included 26 TS patients (16 with a maternally inherited X chromosome and 10 with a paternally inherited X chromosome) and 10 healthy females. They identified 85 differentially expressed genes in TS patients, focusing primarily on gene expression differences between TS patients and controls, with secondary consideration of the parental origin of the X chromosome. The authors further reported that these genes were most significantly associated with the hematological and immune systems [40]. Consistent with their findings, our single-cell RNA sequencing study identified that many of the up-expressed genes in the TS patient are documented to have links to immune responses. Following their approach, we performed additional gene expression analysis of the GEO dataset (GSE46687), identifying 30 overlapping differentially expressed genes consistent with our single-cell RNA sequencing findings, including *XIST*, *TSIX*, *SHROOM1*, *ADTRP*, *JUND*, *SGK1*, *CHKA*, *MAP7D2*, *AIF1*, *ABO*, *OVCH1-AS1*, *GZMB*, *GNLY*, *MYOM2*, *C1orf21*, *FGFBP2*, *CPNE8*, *GZMK*, *LTK*, *MSC-AS1*, *GZMA*, *CCL5*, *SYT11*, *CEBPD*, *PTPRM*, *CST7*, *PZP*, *A2M*, *HPGD*, and *PP2R2B.* Most importantly, *XIST* and *TSIX* emerged as the most significantly down-expressed genes, mirroring the results from our study. While the precise roles of these genes in TS remain unclear, previous research has suggested that many of the 43 identified genes are linked to various immune pathways.

Different innate and humoral immune responses between females and males have been well-documented by others [42]. Females with increased expression of immune-related genes and enhanced immune response are known to be more susceptible to autoimmune diseases and malignancies than males [13]. Female T and B cells have been proposed to explain the increase of female immune response to infection. Among these, CD4(+) T cells are recognized as pivotal contributors to adaptive immunity, playing a crucial role not only in eliminating pathogens, but also in regulating autoimmune diseases and targeting pathogenic cells such as cancer cells [43]. The X chromosome harbors a significant number of genes associated with immune function and thus X chromosome inactivation in females, along with genes evading this inactivation, have been speculated to contribute to the diversity of immune responses [43]. Others have suggested that the major histocompatibility complex (MHC), located in the long arm of the X chromosome, may explain the difference in immunogenic profiles observed in TS [39]. Moreover, some studies suggest that the X chromosome retains genes important for epigenetic regulation [44,45]. Zheng et al. further suggested that expression changes of autosomal genes may be due to the ripple effects of the X chromosome genes through their influence on regulatory expression networks [7]. While the etiology of autoimmunity in TS remains unclear, various differentially expressed genes found in our study appear to be linked to immune responses.

The X chromosome plays a crucial role in ensuring balanced genetic expression between sexes as one X chromosome is randomly silenced through X chromosome inactivation, a process regulated by the X-active specific transcript gene (*XIST*), a non-coding RNA located on the X chromosome. *TSIX*, an antisense RNA to the *XIST* gene, was also down-expressed in TS. Both *XIST* and *TSIX* have been associated with incremental expression levels in X chromosome dosage, when comparing females with 45, X, 46, XX, and 47, XXX karyotypes [46]. These findings suggest that X chromosome dosage may influence various TS phenotypes. In our single-cell RNA sequencing study, the universal expression of the *XIST* gene can function as a positive control, and its lower expression level in TS suggests that X inactivation is absent, validating the accuracy of the research. Furthermore, the lack of X chromosome inactivation in TS may influence autoimmunity, not only through immune-related genes on the X chromosome but also through the lack of X chromosome inactivation and its effects on broader immunogenetic effects [18].

The *SGK1* gene, located on chromosome 6 (6q23.2), encodes serum/glucocorticoid-regulated kinase 1, which has been reported to participate in the development of several human diseases, including cervical cancer, pulmonary fibrosis, Alzheimer’s disease, and type 2 diabetes mellitus [47]. Recent studies have also highlighted its involvement in immune and inflammatory regulatory functions and its role in various diseases such as inflammatory bowel diseases, multiple sclerosis, and sepsis [48]. The *JUND* gene on chromosome 19 (19p13.11) has been reported to regulate lymphocyte proliferation and T helper cell cytokine expression [49]. The *AIF1* gene (6p21.33) is known to be induced by cytokines and interferon to activate macrophage activation and T lymphocytes [50].

Among the up-expressed genes, the *GNLY* gene, located on chromosome 2 (2p11.2), encodes the granulysin that is present in the cytotoxic granules of T cells. Elevated expression of *GNLY* in tissue and serum has been associated with infections, autoimmune disease, transplant rejection, and graft-versus-host reactions. Patients with severe immunodeficiency have been reported to have very low *GNLY* serum levels [51,52]. Additionally, the *GZMB* gene, located on chromosome 14 (14q12), encodes proteins for natural killer (NK) cells and cytotoxic T lymphocytes. These genes, known as cytotoxic genes, are highly expressed in T cells with roles in the immune system. A recent study has suggested that elevated expression of *GNLY* and *GZMB* may facilitate the rapid resolution of SARS-CoV-2 infection by promoting direct cytotoxicity [53]. The *GZMK* and *GZMA* genes, located on chromosome 5 (5q11.2), are also related to cytotoxic effector expression. Granzyme K is believed to be low in cord blood, suggesting that it might be upregulated with immune experience [54]. Granzyme A expression has been associated with cytotoxic activity against tumor or virus-infected cells as well as the stimulation of several immune cell types [55]. The *PPP2R2B* gene, located on chromosome 5 (5q32), has been reported to protect against organ damage caused by activated T cells in chronic inflammation and systemic autoimmune diseases [56]. Additionally, a study has identified this gene as a robust tumor suppressor, playing an important role in anti-tumor immune response. In breast cancer, *PPP2R2B* expression was strongly associated with immune check point inhibitor genes such as *BZMA*, *PRF1*, and *IFNG*, suggesting that downregulation of *PPP2R2B* could take part in tumor immune evasion [57]. The *CCL5* gene located on chromosome 17 (17q12) is known to be involved in immunoregulatory and inflammatory processes [58,59]. *CCL5* is induced during inflammation and plays a crucial role in recruiting activated effector T cells and generating memory T cells [60]. The *CEBPD* gene found on chromosome 8 (8q11.21) encodes CCAAT/enhance-binding protein delta, a key transcription factor that regulates genes involved in immune and inflammatory responses [61]. The *CST7* gene on chromosome 20 (20p11.21) encodes cystatin F protein that regulates the cytotoxicity of natural killer (NK) cells [62]. Previously, *CST7* has also been associated with CD4(+) T cell and CD8 (+) T cell activation in liver cancer [10].

The *MYOM2* gene on chromosome 8 (8p23.3) encodes a protein expressed in cardiac and skeletal muscles. Chen et al. found *MYOM2* to be one of the differentially expressed genes in natural killer cells and that it is enriched in biological pathways associated with HIV replication [63]. The *FGFBP2* gene encodes a protein known to be involved in cytotoxic lymphocyte-mediated immunity (chromosome 4, 4p15.32). The *CPNE8* gene encoding a calcium-dependent protein is on chromosome 12 (12q12). Recent studies have indicated that *CPNE8* is highly correlated with monocytes, macrophages, and neutrophils, suggesting its potential involvement in immune-related pathways [64]. The *LTK* gene on chromosome 15 (15q15.1) encodes leukocyte tyrosine kinase, which is involved in controlling pathways of cell growth and differentiation [65]. Previous studies have found that *LTK* is expressed in B lymphocyte precursor cells, and its gain-of-function is associated with the pathogenesis of systemic lupus erythematosus pathogenesis through upregulating self-reactive B cells [66]. The *PZP* gene encodes PZP protein, which is highly expressed in late pregnancy and plays a role in immune regulation during pregnancy [67]. Located on chromosome 12 (12p13.31), the *PZP* expression level is positively correlated with macrophage and neutrophil levels to regulate the tumor immune microenvironment of hepatocellular carcinoma [68]. The *A2M* gene is located on chromosome 12 (12p13.31) and has been associated with the activation of neutrophil migration, promoting cell division of macrophages and immune-mediated pathways in humans [69]. Further, this gene encodes a protease inhibitor and cytokine transporter involved in proteolysis, as well as cellular immunity and defense mechanisms [70].

Nevertheless, this study has several limitations. Using single-cell RNA sequencing, we identified differentially expressed genes in a Turner syndrome patient, a healthy female volunteer, and a patient with Graves’ disease. In studies involving individuals with rare conditions, the sample size is often limited due to patient availability and financial constraints. While the small sample size may limit broader generalizability and interpretive value, the scRNA-seq technology provides high-resolution gene expression profiling even with limited samples, lending robustness to our findings [71,72]. Furthermore, while the number of genes that differed between the three subjects were relatively small when the top 100 significantly differentially expressed genes were analyzed, genes related to X-inactivation exhibited much lower expression levels in TS, as expected (Supplementary Figure S1A,B). Aside from the prominent X-linked genes, most observed gene expression differences may not be directly attributed to Turner syndrome, as the study does not account for potential variability arising from differences. Nonetheless, despite these limitations, our study highlights distinct and consistent differences in the expression of TS-specific genes such as *XIST* and *TSIX*, which are central to our conclusions. These findings align with the known mechanisms of X chromosome haploinsufficiency in TS, supporting the notion that the observed patterns are not solely attributable to variability among 46,XX individuals. However, while their statistical significance is evident, their clinical significance requires further validation. Additionally, this study focused specifically on CD4(+) T cells from PBMCs to explore differentially expressed genes potentially related to immune function in TS patients. While this approach provided valuable insights, it inherently limited the scope of our findings. Examining a broader range of cell types and tissues would offer a more comprehensive understanding of systemic immune changes in TS. Furthermore, despite our efforts to match the study subjects as closely as possible, there were unavoidable differences in patient characteristics, such as age, height, weight, ABO blood type, and parity. A more controlled study design would strengthen the comparability of future analyses. Nonetheless, many genes identified in our study may be associated with immune responses, although their precise roles in the autoimmune mechanisms of Turner syndrome remain to be clarified. Future studies incorporating larger sample sizes and additional controls will be critical to further validate our findings and enhance their interpretive value. Expanding the analysis to include additional cell types and tissues will also provide more comprehensive insights into the immunogenetic mechanisms underlying TS.

## 5. Conclusions

In this study, we used CD4(+) T cells gated single-cell RNA sequencing of peripheral blood mononuclear cell samples from a Turner syndrome patient, a healthy female, and a patient with Graves’ disease to identify differentially expressed genes. As anticipated, the *XIST* gene was down-expressed in the TS patient (46, X) compared with both the healthy female and the patient with Graves’ disease, who retained two X chromosomes. Thus, our findings may attest for the haploinsufficiency of the X chromosome in the TS patient. Furthermore, we identified 43 overlapping genes in the TS patient compared with the healthy female and the patient with Graves’ disease, many of which were previously linked to immune system functions. While a direct causal inference between these genes and the pathogenesis of TS cannot yet be made, they may serve as potential targets for future studies. These findings offer valuable insights into the differences in genes related to the immune system in TS compared with healthy individuals and those with autoimmune diseases.

## Figures and Tables

**Table 1:** Characteristics of subjects.

	TS	HF	GD
**Subjects**			
Sex	F	F	F
Anthropometric data at blood sampling			
Height (cm)	143	163	162
Weight (kg)	47	55	68
BMI (kg/m^2^)	22.98	20.70	25.91
Age at enrollment (years)	24	43	17
Age at diagnosis (years)	5	N/A	16
Goiter	negative	negative	positive
Free T4 (fT4) at diagnosis, 0.85–1.86 ng/dL	WNL (T4)	WNL	6.38
TSH at diagnosis, 0.17–4.05 mIU/L	WNL	WNL	<0.01
TSHR Ab positive at diagnosis	N/A	negative	positive
Clinically evident TAO (NOSPECS class II or higher), *n* (%)	negative	negative	positive
**Clusters of cells**			
Cluster total	1913	1018	1761
Th2 *GATA3* positive	493	334	441
Estimated number of cells *	2150	1031	1909

Data are presented as mean ± Standard Deviations (SD) or *n* (%). TS, Turner syndrome; HF, healthy female; GD, Graves’ disease; N/A, Not applicable; TSH, thyroid stimulating hormone; TSHR Ab, TSH receptor antibody; TAO, thyroid associated ophthalmopathy; WNL, within normal range. * Estimation by ‘cellranger count’ described in Section 2.

**Table 2:** Significant differences in single-cell gene expression profiles of a patient with TS compared with an HF.

Gene Symbol	Log2 Fold Change (TS/HF)	*p*-Value	Gene Title	Gene Location	Ensembl *
*XIST*	−10.572	3.03 × 10^−106^	X inactive specific transcript	Xq13.2	ENSG00000229807
*OVCH1-AS1*	7.379	1.08 × 10^−35^	OVCH1 antisense RNA 1	12p11.22	ENSG00000257599
*SLC35F1*	4.377	1.25 × 10^−33^	Solute carrier family 35, member F1	6q22.2	ENSG00000196376
*AL592183.1*	6.288	1.57 × 10^−33^	-	-	ENSG00000273748
*FMN1*	4.794	2.26 × 10^−32^	Formin 1	15q13.3	ENSG00000248905
*AC068279.2*	−8.045	2.77 × 10^−31^	-	2p11.2	ENSG00000287763
*MYOM2*	4.921	2.01 × 10^−27^	Myomesin 2	8p23.3	ENSG00000036448
*CALHM6*	−5.436	7.25 × 10^−24^	Family with sequence similarity 26, member F	6q22.1	ENSG00000188820
*MTRNR2L1*	3.478	1.78 × 10^−22^	MT-RNR2-like 1	17p11.2	ENSG00000256618
*GZMH*	5.995	3.83 × 10^−22^	Granzyme H	14q12	ENSG00000100450
*GNLY*	6.773	4.23 × 10^−21^	Granulysin	2p11.2	ENSG00000115523
*SHROOM1*	−3.373	4.42 × 10^−17^	Shroom family member 1	5q31.1	ENSG00000164403
*PPP1R2C*	−7.610	2.88 × 10^−16^	PPP1R2 family member C	Xp11.3	ENSG00000102055
*C1orf21*	5.161	2.68 × 10^−15^	Chromosome 1 open reading frame 21	1q25.3	ENSG00000116667
*ABO*	6.364	4.75 × 10^−15^	ABO, alpha 1-3-N-acetylgalactosaminyltransferase and alpha 1-3-galactosyltransferase	9q34.1	ENSG00000175164
*OVCH1*	5.050	1.54 × 10^−14^	Ovochymase 1	12p11.22	ENSG00000187950
*FGFBP2*	5.269	2.26 × 10^−14^	Fibroblast growth factor binding protein 2	4p15.32	ENSG00000137441
*NKG7*	3.470	2.28 × 10^−14^	Natural killer cell group 7 sequence	19q13.41	ENSG00000105374
*TSIX*	−5.571	2.28 × 10^−14^	TSIX transcript, XIST antisense RNA	Xq13.2	ENSG00000270641
*PLEK*	3.875	1.51 × 10^−13^	Pleckstrin	2p14	ENSG00000115956
*C2orf74*	2.885	1.08 × 10^−12^	Chromosome 2 open reading frame 74	2p15	ENSG00000237651
*AL672277.1*	−6.101	1.20 × 10^−11^	-	Xp22.33	ENSG00000237531
*ADTRP*	−2.401	4.76 × 10^−11^	Androgen-dependent TFPI-regulating protein	6p24.1	ENSG00000111863
*LINC02254*	3.218	6.27 × 10^−11^	-	15q26.2	ENSG00000259664
*SEMA4A*	−2.710	8.19 × 10^−11^	Semaphorin 4A	1q22	ENSG00000196189
*LINC01952*	−2.431	1.47 × 10^−10^	-	7p13	ENSG00000234183
*AC004854.2*	−2.414	1.62 × 10^−10^	-	7p13	ENSG00000272768
*H2AFX*	−1.904	2.24 × 10^−10^	H2A histone family, member X	11q23.3	ENSG00000188486
*FHIT*	−1.850	1.96 × 10^−9^	Fragile histidine triad	3p14.2	ENSG00000189283
*DUSP4*	−2.408	2.22 × 10^−9^	Dual specificity phosphatase 4	8p12	ENSG00000120875

* Ensembl: https://grch37.ensembl.org/Homo_sapiens (accessed on 10 October 2024).

**Table 3:** Significant differences in single-cell gene expression profiles of Th2 cells in a patient with TS compared with an HF.

Gene Symbol	Log2 Fold Change (TS/HF)	*p*-Value	Gene Title	Gene Location	Ensembl *
*XIST*	−8.682	2.00 × 10^−50^	X inactive specific transcript	Xq13.2	ENSG00000229807
*SLC35F1*	4.138	4.76 × 10^−20^	Solute carrier family 35, member F1	6q22.2	ENSG00000196376
*FMN1*	4.792	4.76 × 10^−20^	Formin 1	15q13.3	ENSG00000248905
*OVCH1-AS1*	7.455	1.27 × 10^−17^	OVCH1 antisense RNA 1	12p11.22	ENSG00000257599
*MYOM2*	5.568	3.23 × 10^−16^	Myomesin 2	8p23.3	ENSG00000036448
*AL592183.1*	5.178	1.10 × 10^−15^	-	-	ENSG00000273748
*MTRNR2L1*	3.067	1.81 × 10^−11^	MT-RNR2-like 1	17p11.2	ENSG00000256618
*AC068279.2*	−6.160	6.34 × 10^−9^	-	-	ENSG00000287763
*AC004854.2*	−2.826	1.56 × 10^−7^	-	-	ENSG00000272768
*P2RY8*	−1.898	1.85 × 10^−7^	Purinergic receptor P2Y, G-protein coupled, 8	Xp22.33	ENSG00000182162
*CALHM6*	−4.979	1.08 × 10^−6^	Calcium homeostasis modulator family member 6	6q22.1	ENSG00000188820
*MIDN*	−1.773	6.26 × 10^−6^	Midnolin	19p13.3	ENSG00000167470
*H2AFX*	−1.931	7.41 × 10^−6^	H2A histone family, member X	11q23.3	ENSG00000188486
*AC008569.1*	−1.797	2.83 × 10^−5^	-	-	ENSG00000267379
*PPP1R2C*	−6.345	2.83 × 10^−5^	Protein phosphatase 1, regulatory (inhibitor) subunit 2 pseudogene 9	Xp11.3	ENSG00000102055
*CRYBB2*	−4.295	2.92 × 10^−5^	Crystallin beta B2	22q11.23	ENSG00000244752
*SHROOM1*	−3.324	3.37 × 10^−5^	Shroom family member 1	5q31.1	ENSG00000164403
*SEMA4A*	−2.882	4.87 × 10^−5^	Semaphorin 4A	1q22	ENSG00000196189
*RGCC*	−1.543	6.60 × 10^−5^	Regulator of cell cycle	13q14.11	ENSG00000102760
*ABO*	4.870	7.35 × 10^−5^	ABO, alpha 1-3-N-acetylgalactosaminyltransferase and alpha 1-3-galactosyltransferase	9q34.2	ENSG00000175164
*C2orf74*	2.624	9.18 × 10^−5^	Chromosome 2 open reading frame 74	2p15	ENSG00000237651
*HLA-DRB5*	−2.902	1.75 × 10^−4^	Major histocompatibility complex, class II, DR beta 5	6p21.32	ENSG00000198502
*ADTRP*	−2.487	2.05 × 10^−4^	Androgen-dependent TFPI-regulating protein	6p24.1	ENSG00000111863
*GADD45G*	−2.232	2.18 × 10^−4^	Growth arrest and DNA-damage-inducible, gamma	9q22.2	ENSG00000130222
*GADD45B*	−1.515	2.27 × 10^−4^	Growth arrest and DNA-damage-inducible, beta	19p13.3	ENSG00000099860
*CDKN1A*	−1.819	2.29 × 10^−4^	Cyclin-dependent kinase inhibitor 1A	6p21.2	ENSG00000124762
*LINC02254*	3.420	2.29 × 10^−4^	-	15q26.2	ENSG00000259664
*DUSP4*	−2.390	2.38 × 10^−4^	Dual specificity phosphatase 4	8p12	ENSG00000120875
*DUSP2*	−1.494	3.94 × 10^−4^	Dual specificity phosphatase 2	2q11.2	ENSG00000158050
*AP001160.1*	−1.902	4.38 × 10^−4^	-	-	ENSG00000256690

* Ensembl: https://grch37.ensembl.org/Homo_sapiens.

**Table 4:** Significant differences in single-cell gene expression profiles of a patient with TS compared with a patient with GD.

Gene Symbol	Log2 Fold Change (TS/GD)	*p*-Value	Gene Title	Gene Location	Ensembl *
*XIST*	−10.871	3.82 × 10^−94^	X inactive specific transcript	Xq13.2	ENSG00000229807
*AC105402.3*	−7.110	6.65 × 10^−93^	-	2q23.1	ENSG00000231079
*OVCH1-AS1*	5.334	3.34 × 10^−36^	OVCH1 antisense RNA 1	12p11.22	ENSG00000257599
*MYOM2*	4.690	1.49 × 10^−32^	Myomesin 2	8p23.3	ENSG00000036448
*UTS2*	5.357	2.00 × 10^−32^	Urotensin 2	1p36.23	ENSG00000049247
*HLA-DQB1*	5.452	9.76 × 10^−28^	HLA-DQB1 antisense RNA 1	6p21.32	ENSG00000179344
*CCR6*	3.794	4.83 × 10^−27^	Chemokine (C-C motif) receptor 6	6q27	ENSG00000112486
*PTGER2*	3.658	5.00 × 10^−25^	Prostaglandin E receptor 2	14q22.1	ENSG00000125384
*TSIX*	−6.267	2.33 × 10^−24^	TSIX transcript, XIST antisense RNA	Xq13.2	ENSG00000270641
*AIF1*	−3.035	6.13 × 10^−23^	Allograft inflammatory factor 1	6p21.33	ENSG00000204472
*AL121935.1*	3.828	6.51 × 10^−22^	-	6q27	ENSG00000284825
*NRCAM*	−3.658	1.32 × 10^−20^	Neuronal cell adhesion molecule	7q31.1	ENSG00000091129
*ABO*	6.056	2.16 × 10^−20^	ABO blood group (transferase A, alpha 1-3-N-acetylgalactosaminyltransferase; transferase B, alpha 1-3-galactosyltransferase)	9p34.1	ENSG00000175164
*GZMK*	3.363	7.84 × 10^−20^	Granzyme K	5q11.2	ENSG00000113088
*ARHGEF10*	−3.967	7.84 × 10^−20^	Rho guanine nucleotide exchange factor (GEF) 10	8p23.3	ENSG00000104728
*MYBL1*	3.034	1.05 × 10^−19^	MYB proto-oncogene like 1	8q13.1	ENSG00000185697
*CALHM6*	−4.911	1.15 × 10^−19^	Calcium homeostasis modulator family member 6	6q22.1	ENSG00000188820
*RPS26*	2.641	1.82 × 10^−19^	Ribosomal protein S26	12q13.2	ENSG00000197728
*GREM2*	5.193	3.48 × 10^−19^	Gremlin 2, DAN family BMP antagonist	1q43	ENSG00000180875
*NFKBID*	−3.060	2.13 × 10^−18^	Nuclear factor of kappa light polypeptide gene enhancer in B-cells inhibitor, delta	19q13.12	ENSG00000167604
*EGR1*	−3.644	1.70 × 10^−17^	Early growth response 1	5q31.2	ENSG00000120738
*MIR155HG*	−3.011	1.78 × 10^−17^	MIR155 host gene	21q21.3	ENSG00000234883
*RASGRP3*	−3.044	1.93 × 10^−17^	RAS guanyl releasing protein 3	2p22.3	ENSG00000152689
*MTRNR2L1*	2.647	3.63 × 10^−17^	MT-RNR2-like 1	17p11.2	ENSG00000256618
*SGK1*	−2.563	9.54 × 10^−17^	Serum/glucocorticoid regulated kinase 1	6q23.2	ENSG00000118515
*TAF4B*	−2.547	1.56 × 10^−16^	TATA-box binding protein associated factor 4b	18q11.2	ENSG00000141384
*GNLY*	4.384	2.02 × 10^−16^	Granulysin	2p11.2	ENSG00000115523
*CFH*	3.345	2.33 × 10^−16^	Complement factor H	1q31.3	ENSG00000000971
*AC103591.3*	−2.648	9.13 × 10^−16^	-	1p31.1	ENSG00000273338
*HLA-DQA2*	6.853	9.58 × 10^−16^	Major histocompatibility complex, class II, DQ alpha 2	6p21.32	ENSG00000237541

* Ensembl: https://grch37.ensembl.org/Homo_sapiens.

**Table 5:** Significant differences in single-cell gene expression profiles of Th2 cells in a patient with TS compared with a patient with GD.

Gene Symbol	Log2 Fold Change (TS/GD)	*p*-Value	Gene Title	Gene Location	Ensembl *
*AC105402.3*	−7.194	4.36 × 10^−60^	-	-	ENSG00000231079
*XIST*	−9.058	1.75 × 10^−47^	X inactive specific transcript	Xq13.2	ENSG00000229807
*MYOM2*	5.730	1.16 × 10^−19^	Myomesin 2	8p23.3	ENSG00000036448
*OVCH1-AS1*	6.255	5.39 × 10^−19^	OVCH1 antisense RNA 1	12p11.22	ENSG00000257599
*AIF1*	−3.349	1.46 × 10^−16^	Allograft inflammatory factor 1	6p21.33	ENSG00000204472
*CCR6*	3.846	8.59 × 10^−16^	Chemokine (C-C motif) receptor 6	6q27	ENSG00000112486
*RPS26*	2.624	4.35 × 10^−15^	Ribosomal protein S26	12q13.2	ENSG00000197728
*UTS2*	5.219	2.28 × 10^−14^	Urotensin 2	1p36.23	ENSG00000049247
*MIR181A1HG*	−3.142	6.48 × 10^−12^	MIR181A1 host gene	1q32.1	ENSG00000229989
*GREM2*	6.964	1.42 × 10^−11^	Gremlin 2, DAN family BMP antagonist	1q43	ENSG00000180875
*ARHGEF10*	−4.897	1.99 × 10^−11^	Rho guanine nucleotide exchange factor (GEF) 10	8p23.3	ENSG00000104728
*MYBL1*	3.148	1.99 × 10^−11^	MYB proto-oncogene like 1	8q13.1	ENSG00000185697
*PTGER2*	3.031	6.15 × 10^−11^	Prostaglandin E receptor 2	14q22.1	ENSG00000125384
*MTRNR2L1*	2.645	1.69 × 10^−10^	MT-RNR2-like 1	17p11.2	ENSG00000256618
*HLA-DQB1*	5.067	1.78 × 10^−10^	Major histocompatibility complex, class II, DQ beta 1	6p21.32	ENSG00000179344
*NFKBID*	−3.401	1.78 × 10^−10^	NFKB inhibitor delta	19q13.12	ENSG00000167604
*WHAMM*	−2.236	2.62 × 10^−10^	WAS protein homolog associated with actin, golgi membranes and microtubules	15q25.2	ENSG00000156232
*TSIX*	−6.009	3.04 × 10^−10^	TSIX transcript, XIST antisense RNA	Xq13.2	ENSG00000270641
*NRCAM*	−3.700	4.58 × 10^−10^	neuronal cell adhesion molecule	7q31.1	ENSG00000091129
*EGR1*	−3.853	4.73 × 10^−10^	Early growth response 1	5q31.2	ENSG00000120738
*RASGRP3*	−2.966	6.59 × 10^−10^	RAS guanyl releasing protein 3	2p22.3	ENSG00000152689
*SGK1*	−2.434	1.01 × 10^−9^	Serum/glucocorticoid regulated kinase 1	6q23.2	ENSG00000118515
*AC103591.3*	−2.805	3.14 × 10^−9^	-	-	ENSG00000273338
*AL121935.1*	3.566	7.31 × 10^−9^	-	-	ENSG00000284825
*TAF4B*	−2.438	7.72 × 10^−9^	TATA-box binding protein associated factor 4b	18q11.2	ENSG00000141384
*CHKA*	−2.280	1.20 × 10^−8^	Choline kinase alpha	11q13.2	ENSG00000110721
*CRIP2*	2.598	1.20 × 10^−8^	Cysteine-rich protein 2	14q32.33	ENSG00000182809
*AC008569.1*	−2.192	1.20 × 10^−8^	-	-	ENSG00000267379
*JUN*	−1.974	2.06 × 10^−8^	Jun proto-oncogene	1p32.1	ENSG00000177606
*EZH2*	−2.361	3.66 × 10^−8^	Enhancer of zeste homolog 2	7q36.1	ENSG00000106462

* Ensembl: https://grch37.ensembl.org/Homo_sapiens.

## Data Availability

All the data analyzed in this study are not publicly available for the privacy of the research participants but are available from the corresponding author upon reasonable request.

## Supplementary Materials

The following supporting information can be downloaded at: https://www.mdpi.com/article/10.3390/cells14020093/s1, Table S1: Significantly up expressed genes in TS compared with HF; Table S2: Significantly down expressed genes in TS compared with HF; Table S3: Significantly up expressed genes in TS compared with GD; Table S4: Significantly down expressed genes in TS compared with GD; Figure S1: (A) The triangle diagram illustrating the number of genes with significant differential expression between each pair of subjects (Turner syndrome, TS; Graves’ disease patient, GD; healthy female, HF); (B) The radar plots depicting the relative expression levels of the 43 genes with the most significant differences in a patient with TS compared with both an HF and a patient with GD. The expression values are normalized and plotted on a scale from 0 to the maximum expression level observed across the three subjects.
