# Supplementary material for: Immune Gene Expression Profiling in Individuals with Turner Syndrome, Graves’ Disease, and a Healthy Female by Single-Cell RNA Sequencing: A Comparative Study
Source: Cells. 2025 Jan 10;14(2):93. doi: 10.3390/cells14020093 (PMC11764232; doi:10.3390/cells14020093)

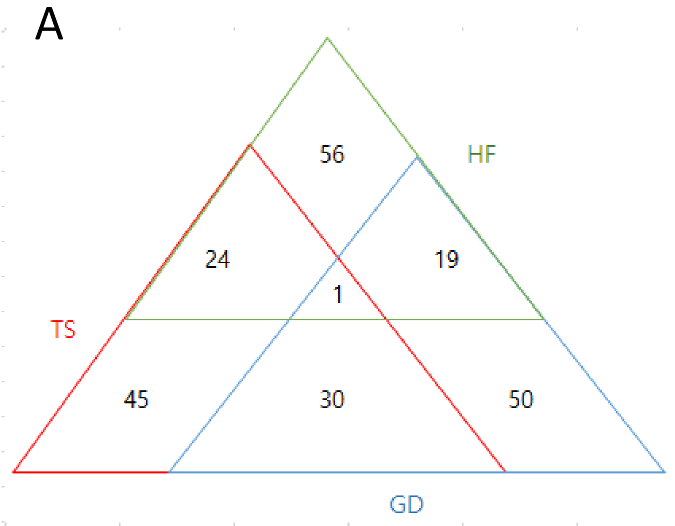

Supplementary Figure A. The triangle diagram illustrating the number of genes with significant differential expression between each pair of subjects (Turner Syndrome, TS; Graves' Disease patient, GD; Health Female, HF) ; **Supplementary Figure B.** The radar plots depicting the relative expression levels of the 43 genes with the most significant differences in a patient with TS compared to both a HF and a patient with GD. The expression values are normalized and plotted on a scale from 0 to the maximum expression level observed across the three subjects.

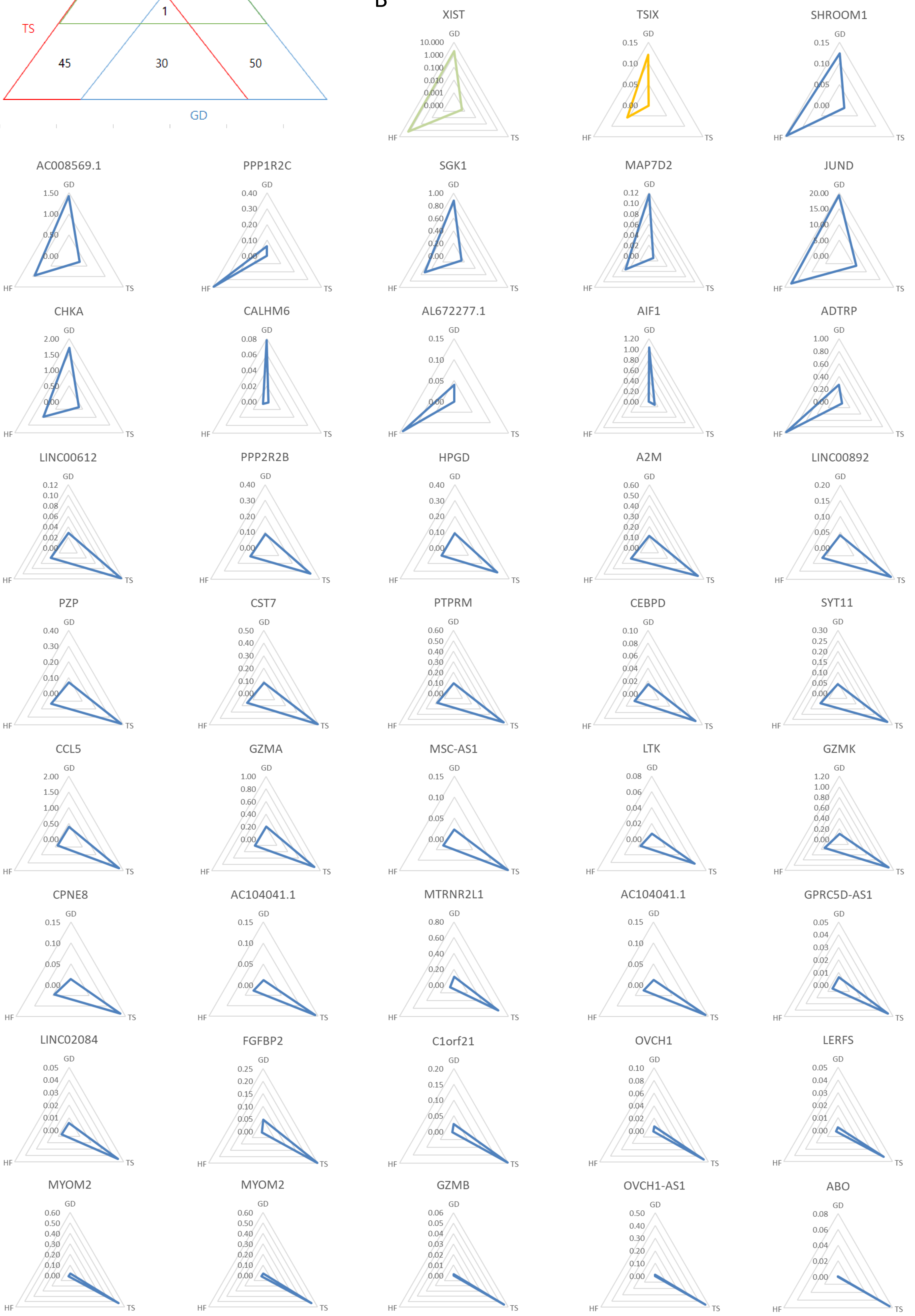

Supplement: Supplementary file 1 [file cells-14-00093-s001.zip › Figure S1.pdf]
